# The Effect of Fetal Bovine Acellular Dermal Matrix Seeded with Wharton’s Jelly Mesenchymal Stem Cells for Healing Full-Thickness Skin Wounds

**DOI:** 10.3390/genes14040909

**Published:** 2023-04-13

**Authors:** Reyhaneh Nassiri Mansour, Elham Hasanzadeh, Mozhgan Abasi, Mazaher Gholipourmalekabadi, Amir Mellati, Seyed Ehsan Enderami

**Affiliations:** 1 Department of Tissue Engineering and Regenerative Medicine, School of Advanced Technologies in Medicine, Mazandaran University of Medical Sciences, Sari 4815733971, Iran; r.nasiri@mazums.ac.ir (R.N.M.);; 2 Cellular and Molecular Research Center, Iran University of Medical Sciences, Tehran 1449614535, Iran; 3 Department of Tissue Engineering & Regenerative Medicine, Faculty of Advanced Technologies in Medicine, Iran University of Medical Sciences, Tehran 1449614535, Iran; 4 Department of Medical Biotechnology, Faculty of Allied Medicine, Iran University of Medical Sciences, Tehran 1449614535, Iran; 5 Molecular and Cell Biology Research Center, Faculty of Medicine, Mazandaran University of Medical Sciences, Sari 4815733971, Iran; 6 Immunogenetics Research Center, Department of Medical Biotechnology, School of Advanced Technologies in Medicine, Mazandaran University of Medical Sciences, Sari 4815733971, Iran

**Keywords:** acellular dermal matrix, Wharton’s jelly mesenchymal stem cells, skin wounds, wound healing, regenerative medicine

## Abstract

The treatment of full-thickness skin wounds is a problem in the clinical setting, as they do not heal spontaneously. Extensive pain at the donor site and a lack of skin grafts limit autogenic and allogeneic skin graft availability. We evaluated fetal bovine acellular dermal matrix (FADM) in combination with human Wharton’s jelly mesenchymal stem cells (hWJ-MSCs) to heal full-thickness skin wounds. FADM was prepared from a 6-month-old trauma-aborted fetus. WJ-MSCs were derived from a human umbilical cord and seeded on the FADM. Rat models of full-thickness wounds were created and divided into three groups: control (no treatment), FADM, and FADM-WJMSCs groups. Wound treatment was evaluated microscopically and histologically on days 7, 14, and 21 post-surgery. The prepared FADM was porous and decellularized with a normal range of residual DNA. WJ-MSCs were seeded and proliferated on FADM effectively. The highest wound closure rate was observed in the FADM-WJMSC group on days 7 and 14 post-surgery. Furthermore, this group had fewer inflammatory cells than other groups. Finally, in this study, we observed that, without using the differential cell culture media of fibroblasts, the xenogeneic hWJSCs in combination with FADM could promote an increased rate of full-thickness skin wound closure with less inflammation.

## 1. Introduction

The skin, as the largest organ, covers the whole body and can protect us from the effects of environmental hazards. Any defects in the structure and integrity of the skin lead to wound formation [1]. Trauma, burns, surgical interventions, cutaneous malignancies, and systemic diseases such as diabetes cause wounds that could be acute or chronic in terms of their healing time frame [2,3]. According to the depth and area of the defect, wounds could be classified into partial-thickness or full-thickness wounds [4]. Due to the loss of both the epidermis and dermis in full-thickness wounds, the skin is unable to regenerate spontaneously. Therefore, a lack of skin epithelialization leads to extensive scar tissue formation at the defect site. These large wounds cannot be healed through conventional surgical methods [5]. The use of autologous skin grafts is considered the standard treatment strategy for these wounds. Split-thickness and full-thickness grafts can result in unpleasant appearances or skin functional defects on the lesion site [6]. While it is the standard treatment, it is a painful procedure and may leave extensive scarring at the donor site [7]. It has also been shown that xenogeneic skin substitutes can cover these wounds; however, there is a risk of microbial infection and cell rejection by the host immune system [8]. Tissue-engineered constructs are another kind of structure that can be used for wound coverage.

Tissue-engineered constructs contain a supportive scaffold for transplanting cells. This cell scaffold structure is an alternative coverage for full-thickness wound healing [9]. The scaffold can be made out of synthetic or natural polymers as well as tissue-derived acellular matrices [10]. The natural scaffolds (dermal substitutes) have more biocompatibility as their structures and compositions are more similar to the natural extracellular matrix (ECM) than other structures. Thereby, they can provide a better microenvironment for cellular functions [9]. It has been reported that dermal substitutes have an increased ability to support cell attachment, infiltration, proliferation, function, and the regeneration of different skin layers [11,12,13]. Allograft or xenograft acellular dermal matrix (ADM) has been used for soft tissue regeneration with promising outcomes [14,15]. ADM is currently used as a medical implement for wound healing and skin regeneration. It is considered one of the best skin substitutes for its elasticity, absorption of exodus wound secretions, integrity with the wound bed, and ability to impede microorganisms [16].

Mesenchymal stem cells (MSCs) are extracted from various sources and are ideal for skin regeneration due to their immunomodulatory effects. MSCs from Wharton’s jelly of the umbilical cord (WJ-MSCs) have attracted attention as an allogeneic use for skin regeneration [17]. Previous works have shown that WJ-MSCs could heal wounds by regulating inflammation [17,18,19]. However, the local transplantation of MSCs causes the low retention of cells at damaged sites, leading to slow recovery [20]. As a result, researchers propose the immobilization of MSCs on matrices (i.e., fabricated scaffolds or acellular natural matrices) before transplantation at wound sites [18,21,22]. During the regeneration process, differentiation growth factors such as fibroblast growth factor (FGF) and keratinocyte growth factor (KGF) are the most important factors in skin regeneration. Therefore, the use of these specific growth factors is common in the healing process and regeneration of the skin. As other studies have shown, the extreme and uncontrolled use of these factors can cause the formation of scar tissue and even some malignancy later in the life of the treated person. Therefore, it is necessary to have a limit regarding the use of growth factors. In our study, we want to achieve a reliable and safe method to prepare a tissue-engineered construct by culturing WJ-MSCs on fetal bovine ADM for healing the full-thickness skin wounds of animal models without any specific or differential growth factors. Although the use of allograft ADM is limited due to the lack of skin donors, we used xenograft ADM derived from fetal bovine as a supportive scaffold for WJ-MSCs. 

## 2. Materials and Methods

### 2.1. Preparation of Acellular Dermal Matrices

In this study, fetal bovine acellular dermal matrix (FADM) was prepared as described by Gaucherand Jarraya [23]. Fresh skin from a 6-month-old trauma-aborted fetus was collected from a local slaughterhouse (Dashtenaz, Sari, Iran). The abdominal zone of the skin was cut and then washed with distilled water to remove excess blood. Thereafter, 10 × 10 cm pieces of the skin were prepared using a sharp blade. After shaving the skin hair with a safety razor, the dermal layer with a 0.8–1 mm thickness was separated from the hypodermal and epidermal layers mechanically. The skin was stretched and placed on a plate from the epidermal side. The skin was held tightly on the plate. By drawing a leather skiver over the tissue, the hypodermal layer was separated. In the same manner, the epidermis was removed from the epidermal side of the skin. The dermal matrix was immersed in a 20% sodium hypochlorite (NaOCl, Dr. Mojallali Co., Tehran, Iran) solution for 30 min on a shaker. After washing the matrix, the sub-dermal flesh and hypodermis were discarded by drawing a flat blade on the surface of the matrix. Afterward, a 0.52 N NaOH (Dr. Mojallali Co., Tehran, Iran) solution was added to the tissue for 15 min at 4 °C. After applying mechanical pressure on the surface of the tissue, this step was repeated once more. The tissue was immersed in a descending concentration series of chloroform (Dr. Mojallali Co., Tehran, Iran) and ethanol/water (70%) mixture solutions (3:1, 2:1, and 1:1 ratio) to remove adipose tissue. This was followed by immersing the tissues in an ascending concentration series of ethanol (Dr. Mojallali Co., Tehran, Iran) solutions (70%, 80%, 90%, 95%, and 100%) to remove the water. The samples were immersed in phosphate-buffered saline (PBS, Gibco, Watlham, MA, USA) and then lyophilized. 

### 2.2. DNA Quantification

The amount of residual DNA in the ADM samples was measured by the FavorPrep™ Tissue Genomic DNA Extraction mini kit (Favorgen Biotech Corp, Ping-Tung, Taiwan). The DNA extraction included the lysis of the sample (ADM), DNA collection, and purification. The DNA concentration was measured using a UV–Vis Spectrophotometer (Thermofisher, Watlham, MA, USA) at 260 nm and 280 nm optical densities. The fresh fetal bovine skin was also used as a reference, as its DNA concentration was considered 100%. 

### 2.3. Collagen Content Analysis

Both native and decellularized dermal matrices were evaluated by a collagen assay kit (Biocolor, Carrickfergus, UK) for their collagen content. In this regard, the hydroxyproline content, as an indicator of collagen, was determined in a colorimetric assay. The samples were immersed in a solution containing acetic acid (0.5 M) and pepsin (1 mg/mL (*w*/*v*) (Sigma-Aldrich, St. Louis, MO, USA) overnight. After sample solubilization, the lysates were treated with Sircol dye for 30 min at RT. Thereafter, the OD of samples was determined at 555 nm. 

### 2.4. Glycosaminoglycan Evaluation

The level of glycosaminoglycans (GAGs) in the native and decellularized dermal matrices was assessed by a Blycan assay kit (Biocolor, Carrickfergus, UK) according to the kit’s instructions. The values were expressed as μg/mg of GAGs per wet dermal matrix tissue weight. 

### 2.5. Isolation and Expansion of MSCs from Wharton’s Jelly

The MSCs were isolated from Wharton’s jelly of the human umbilical cord. Briefly, cord samples in a PBS solution containing 3X antibiotics (penicillin/streptomycin) and a 3X antifungal drug (amphotericin B) were transferred to the laboratory. The samples were washed with the PBS/antibiotics/antifungal solution several times to eliminate potential microbial contaminations and fragment blood and bloodless residues. After separating blood vessels, the Wharton jelly was isolated and crushed. After washing with PBS, Wharton jelly pieces were cultured in high glucose DMEM medium (Nita Fan Co., Sari, Iran) containing 20% FBS (Gibco, Grand Island, NE, USA) and incubated at 37 °C in 5% CO_2_ and 90% humidity. After several days, while the stem cells spread on the plate surface, the detached Wharton jelly pieces were removed. At 80% confluency, the cells were trypsinized with trypsin-EDTA (Gibco, Grand Island, NE, USA) and passaged. The isolated stem cells were observed under an inverted microscope to evaluate the stem cell morphology as well as to ensure there was no microbial contamination. 

### 2.6. Stem Cell Characterization by Flow Cytometry

The extracted WJ-MSCs were cultured in a DMEM/F12 medium containing 10% FBS. The cells in passage 3 were detached by trypsin–EDTA, centrifuged for 5 min at 1200 rpm, and resuspended in human serum. The cells were kept at 4 °C for 30 min and centrifuged for 5 min at 1200 rpm. At this time, the cells (pellet) were resuspended in 3% *v*/*v* goat serum in PBS. The cells were incubated with CD34, CD166, and CD105phycoerythrin (PE)-conjugated, and CD45, CD90, and CD73 fluorescent isothiocyanate (FITC)-conjugated mouse anti-human antibodies on ice for 1 h. The cells were washed in PBS and centrifuged at 12,000 rpm for 5 min. Finally, the cells were resuspended in PBS and analyzed by flow cytometry (Attune Acoustic Focusing Cytometer). The quantification of CD marker expression was measured using FlowJo software version 7.3. 

### 2.7. Multipotency of WJ-MSCs

To further characterize the extracted WJ-MSCs, the cells were cultured in osteogenic and adipogenic media to evaluate their multipotency. The osteogenic medium included high-glucose DMEM and 10% FBS supplemented with dexamethasone, ascorbic acid, and β-glycerophosphate (all from Gibco). The adipogenic medium included high-glucose DMEM and 10% FBS supplemented with dexamethasone, indomethacin, and 2 µmol insulin (all from Gibco). After 21 days of culture, the cells were stained with Alizarin Red or Oil Red O to evaluate the osteogenesis and adipogenesis of the MSCs. 

### 2.8. Cell Proliferation and Penetration Assay

The ability of the WJ-MSCs attachment and proliferation on FADM was assessed by 3-(4,5-dimethyl-thiazolyl-2)-2,5-diphenoltetrazolium bromide (MTT) assay. Briefly, WJ-MSCs with the concentration of 5 × 10^3^ cells per cm^2^ were cultured on each sterilized FADM in a 24-well plate and incubated for 1, 3, and 5 days at 37 °C and 5% CO_2_. At the selected time points, MTT (Gibco, Grand Island, NE, USA) solution (5 mg/mL in DMEM) was added to each well, and the plate was incubated for a further 3 h. Thereafter, the formazan crystals were solubilized in dimethylsulfoxide (DMSO). To evaluate cell proliferation potential, the optical density at 570 nm was measured by a microplate reader (BioTek Instruments Inc, Winooski, VT, USA). 

To visualize the microstructure, small pieces of FADM (before and after cell seeding) were prepared and immersed in liquid nitrogen. Samples were cross-sectioned and mounted on an aluminum stud. The surface of the samples was sputter-coated with gold nanoparticles. The samples were observed under a scanning electron microscope (JEOL, Paris, France). 

To evaluate the penetration of the stem cells in FADM, the matrices were fixed in 10% (*w*/*v*) formalin (Sigma-Aldrich, St. Louis, MO, USA) after cell seeding. The matrices were dehydrated, paraffin-embedded, and sectioned into 5 μm thick samples using a microtome device. Then the samples were deparaffinized, rehydrated, and stained by 4, 6-diamidino-2-phenylindole (DAPI; Thermo Fisher Scientific, Watlham, MA, USA). The attachment, distribution, and penetration of the WJ-MSCs were evaluated under the light microscope.

### 2.9. RNA Extraction and Quantitative Real-Time Polymerase Chain Reaction

WJ-MSCs and FADM-WJ-MSCs were cultured in a standard medium for 7 days. To extract the total RNA from the matrix, RNX-Plus solution (EX6101, Sinaclon, Tehran, Iran) was used, and the quantity and quality of the RNA were assessed using a UV–Vis Spectrophotometer (Thermofisher, Watlham, MA, USA) at 260 nm and 280 nm optical densities. Thereafter, the extracted RNA was turned into complementary DNA (cDNA) using PrimeScript™ RT reagent kit (TaKaRa, Japan). The relative expression of *VEGFa*, *bFGF*, and *IL-1β* was evaluated by SYBR Premix Ex Taq™ (TliRNaseH Plus, TaKaRa, Japan) PCR kit while hypoxanthine-guanine phosphoribosyl transferase (*Β-actin*) was used as an internal control. The primer sequences are provided in Table 1. The PCR reaction was performed using a StepOne™ (Applied Biosystem, CA, USA) machine.

### 2.10. In Vivo Implantation of the ADMs

The animal experiments were performed after approval by the Ethics Committee of Mazandaran University of Medical Sciences. In this study, 48 rats (male and female) with 250–300 g weight and 3–4 months old were used. The animals were kept in a room with standard conditions, including a room temperature of 20–25 °C. All animals received water and a regular rat diet. The animal model of skin injury was prepared as described by Mendonca and coworkers [24]. Briefly, the anesthetization of the animals was performed by a single dose of intraperitoneal (i.p) injection of ketamine (50 mg/kg) and xylazine (10 mg/kg) (Alfasan, Utrecht, The Netherlands). The skin was prepared for surgery after hair shaving and skin disinfection with ethanol (70%) and iodine. Afterward, with disinfected scissors, a full-thickness wound with a 1.5 × 2 cm dimension was made on the back of each rat. The rat models of the skin wound were randomly divided into three groups, each with eight rats. Group 1 received no treatment option (control). In groups 2 and 3, the wound was covered with FADM or FADM-WJMSCs. No other treatment option was used during the healing phase. On days 7, 14, and 21 post-surgery, colored photographs were taken using a digital camera. These photographs were imported into ImageJ software (version 1.8.0), and wound closure was calculated as follows [25]:

(1) $$\mathrm{Wound}\;\mathrm{closure}\;\left(\%\right)=\frac{A_{0}-A}{A_{0}}\times 100$$

A_0_ is the area of the wound at the initial time point, and A is the area of the wound at each postoperative time point. 

### 2.11. Histological Staining

On days 7, 14, and 21 post-treatment, three rats in each study group were randomly selected and euthanized. In each rat, the skin of the wounded site and its surrounding skin were picked and immersed in a 10% formalin (Dr. Mojallali Co., Tehran, Iran) solution with a pH of 7.26. After 48 h, the specimens were dehydrated in an ascending concentration series of ethanol and paraffin-embedded. Then, samples of 5 μm thickness were prepared using a microtome device. After staining in hematoxylin and eosin (Neutron^®^ Pharmachemical Co., Tehran, Iran), the sections were evaluated by a camera-equipped light microscope (Olympus BX51, Olympus, Tokyo, Japan) for inflammation, epithelialization, and tissue granulation. 

### 2.12. Statistical Analysis

As the measurements were carried out in triplicate, the mean and standard deviation (SD) of each measurement were calculated and recorded. The data were analyzed by SPSS version 23.0 software using *t*-tests and one-way analysis of variance (ANOVA) with *p* < 0.05 values considered statistically significant.

## 3. Results

### 3.1. Characterization of ADM

**Figure 1:**
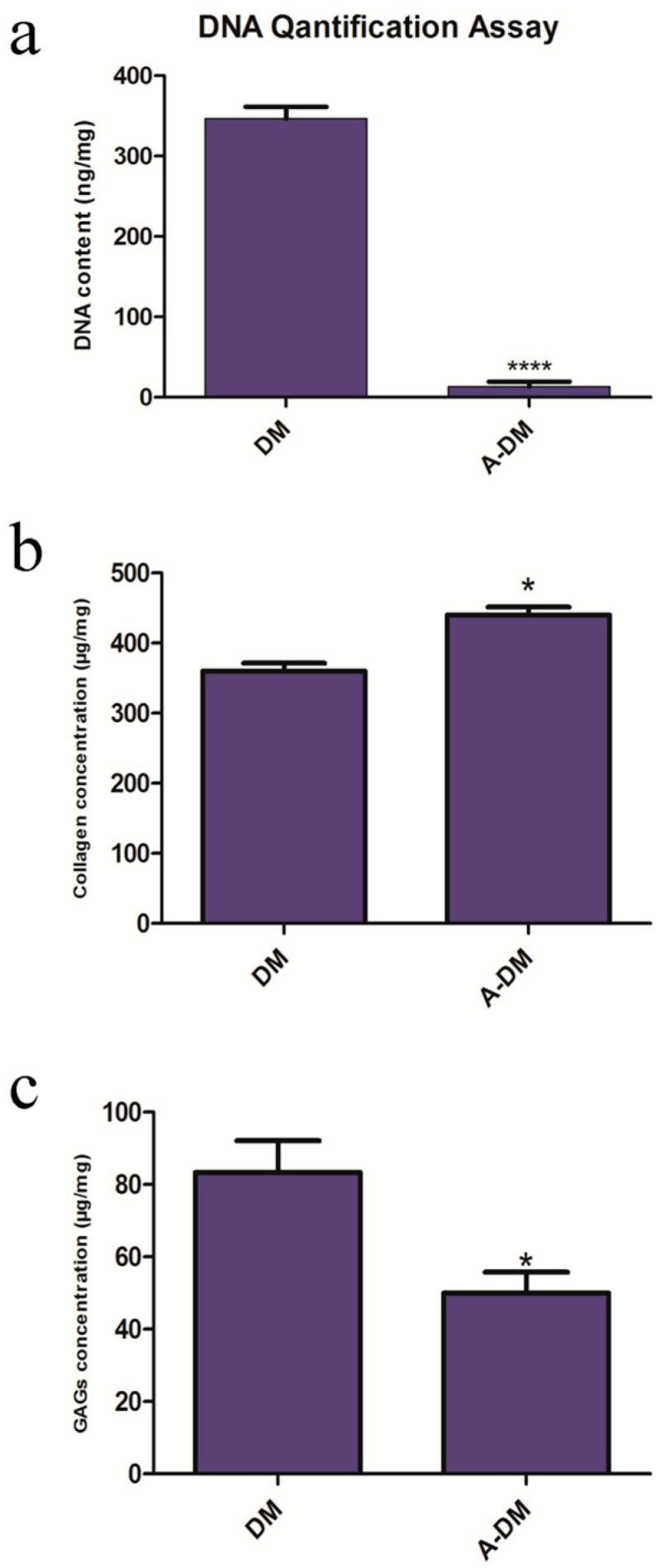
FADM characterization. DNA content dramatically decreased after decellularization of the matrix (**** *p* < 0.0001) (**a**). Higher collagen concentration (**b**) and lower GAGs content (**c**) were observed in FADM (* *p* < 0.05).

The results of the DNA quantification are provided in Figure 1a, which indicates that the quantity of DNA reduced dramatically after decellularization. While the DNA content was 341.3 ± 17.1 ng/mg for the dried native dermis, it was 5.7 ± 1.1 ng/mg for dried FADM (**** *p* < 0.0001). The collagen concentration in both native and decellularized matrices is shown in Figure 1b. While the collagen content was 361 ± 10.4 µg/mg for the native matrix, the value was 441 ± 11.7 µg/mg for the decellularized matrix (* *p* < 0.05). Figure 1c also shows the GAGs content for native and decellularized matrices. As shown, the GAGs concentration is significantly lower in the decellularized matrix when compared to the native matrix (* *p* < 0.05). These results indicate that the decellularization method was effective for the preparation of FADM with favorable properties for downstream experiments. In addition, we previously reported that this decellularization method provided a matrix with favorable mechanical and thermal properties [25].

### 3.2. Characterization of the WJ-MSCs

**Figure 2:**
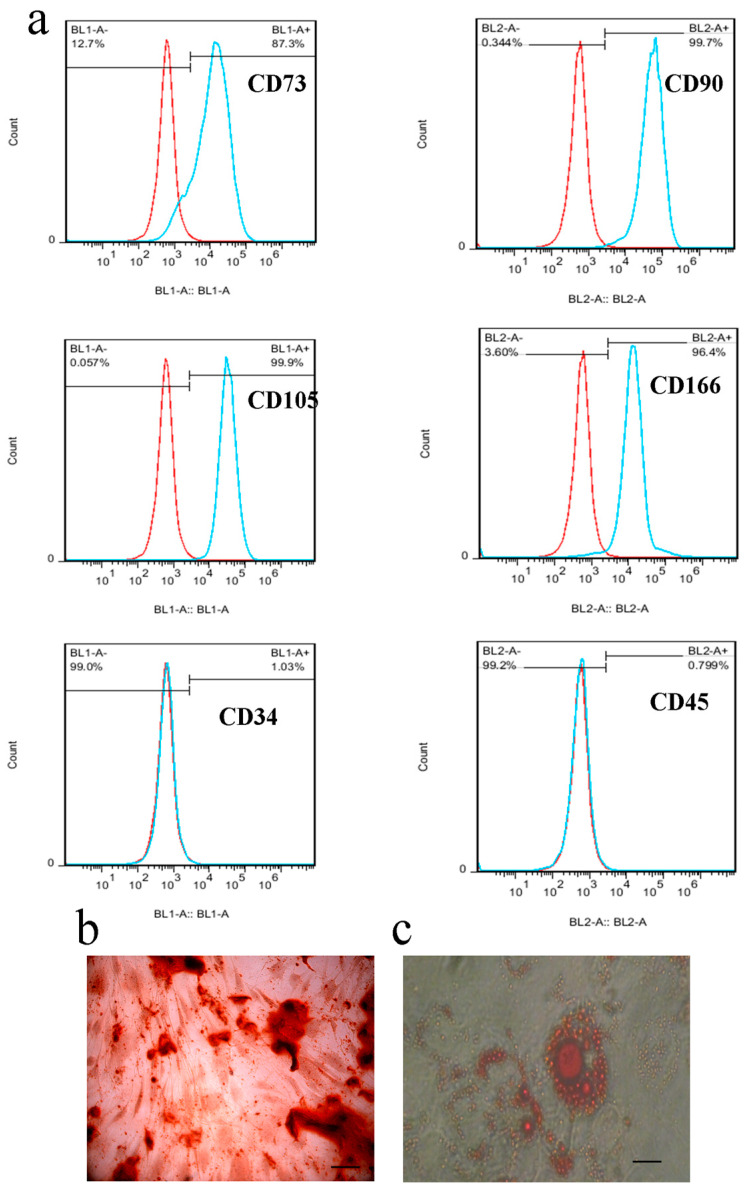
WJ-MSCs characterization. The expression levels of CD34, CD166, CD105, CD45, CD90, and CD73 markers were observed in isolated WJ-MSC. There are two peaks on the histogram graph: cell-positive (blue line) and cell-negative (red line) peak. (**a**) The reddish calcium deposits (**b**) and reddish oil droplets (**c**) were observed after staining of differentiated cells with alizarin red staining and Oil Red O staining, respectively.

The isolated stem cells have shown to be spindle shape when attached to adherent culture flasks. The results of flowcytometry are illustrated in Figure 2a. Isolated WJ-MSCs were evaluated for the expression of CD34, CD166, CD105, CD45, CD90, and CD73 markers, indicating that the isolated cells express multipotency markers. Flow cytometry data displayed that cell surface-specific markers such as CD73 (87.3%), CD90 (99.7%), CD166 (96.4%), and CD105 (99.9%) have very high expression, while CD45 (0.79%) and CD34 (1.03%) markers have very low expression. The osteogenic and adipogenic potential of the isolated WJ-MSCs is illustrated in Figure 2b,c. After three weeks of culture in the osteogenic medium, the alizarin red staining showed reddish calcium deposits in cultured cells. In addition, the Oil Red O staining indicated that the extracted WJ-MSCs were differentiated into adipose-like cells containing reddish oil droplets in the cytoplasm after three weeks of induction. 

### 3.3. Cell Proliferation and Penetration

**Figure 3:**
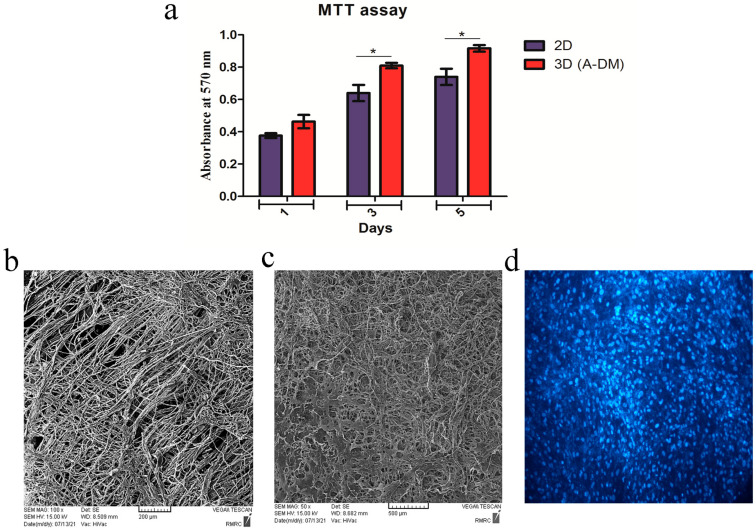
Cell proliferation and penetration in FADM. (**a**) The result of the MTT assay shows that cell proliferation is significantly higher in the FADM group than in 2D tissue culture plates (TCPs) on days 3 and 5 of culture (* *p* < 0.05). (**b**) SEM photograph shows the unidirectionally aligned collagen fibers. (**c**) SEM photograph shows the presence and adhesion of MSCs on the surface of FADM. (**d**) DAPI staining shows the presence of MSCs on the surface of FADM.

The proliferation ability of the WJ-MSCs on FADM was evaluated by MTT assay. The results are presented in Figure 3a. As shown, a higher number of WJ-MSCs were observed on day 5 in comparison to days 1 and 3 in both the 2D and FADM groups. This shows that the cells were able to attach to FADM and proliferate. In addition, a higher proliferation of WJ-MSCs was observed in the FADM group, indicating that the prepared matrix is biocompatible and favorable for cell adhesion and proliferation. The microstructure of the prepared FADM, before and after cell seeding, was qualitatively evaluated by SEM. The photographs taken from the superficial and cross-section sides are provided in Figure 3b,c. As shown, FDAM contains discrete and intact collagen fibers. In addition, the photographs show that these fibers are aligned unidirectionally. To evaluate the potential of the WJ-MSCs to penetrate into FADM, the matrix was stained by DAPI. The results show the potential of the WJ-MSCs to attach to, penetrate, and grow on the prepared 3D matrix (Figure 3d). 

### 3.4. Gene Expression Results

**Figure 4:**
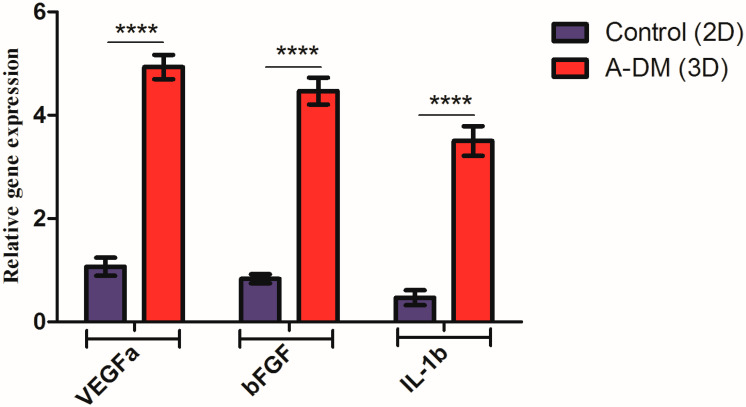
In vitro angiogenesis assay. A higher expression of VEGFa, bFGF, and IL_1β observed in the FADM-WJMSC group (**** *p* < 0.0001).

The angiogenesis potential of the FADM-WJMSCs was evaluated in vitro. The results are illustrated in Figure 4. As shown, after 7 days of culture, the expression of VEGFa, bFGF, and IL-1β was significantly higher in the FADM-WJMSCs group than the control (**** *p* < 0.0001). This indicates that FADM-WJMSCs may improve wound healing by inducing neovascularization.

### 3.5. Wound Closure

**Figure 5:**
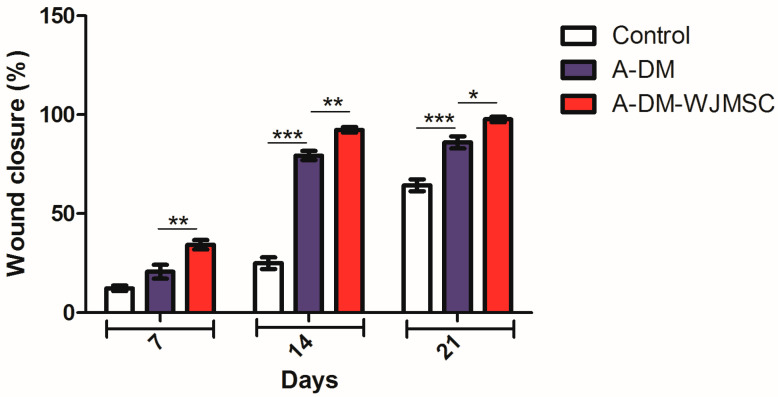
Wound closure rates were quantitatively calculated by image analysis of photographs at different time points (* *p* < 0.05, ** *p* < 0.01, *** *p* < 0.001).

The wound closure results at days 0, 7, 14, and 21 post-surgery were calculated and presented in Figure 5. The wound closure was 12.1%, 23.4%, and 34.21% for day 7 post-surgery in the control, FADM, and FADM-WJMSC groups, respectively. The values were 29%, 88.2%, and 94.1% for day 14 post-surgery, and 61.1%, 91.6%, and 100% for day 21 post-surgery. As shown, wound closure increased over time in all three groups. The results also show that wound closure was higher in the FADM group than the control group and higher in the FADM-WJMSC group than the FADM group at days 7 and 14 post-surgery (* *p* < 0.05). On day 21 post-surgery, almost complete wound closure was observed in the FADM-WJMSC group, indicating the potential of FADM in combination with WJ-MSCs for skin regeneration at the full-thickness wound site. 

### 3.6. Histological Studies

**Figure 6:**
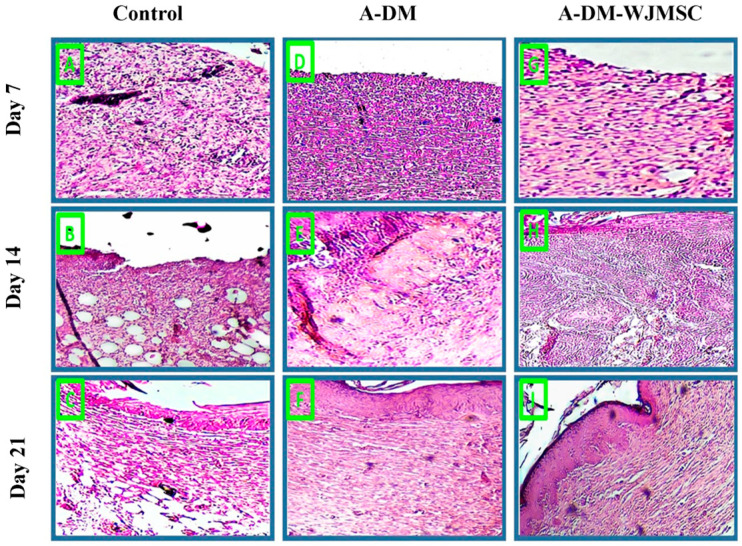
Histological evaluation of skin biopsies using H&E staining for control (untreated), FADM, and FADM-WJ-MSC groups at 7, 14, and 21 days after implantation. The images (**A**–**C**), (**D**–**F**), and (**G**–**I**) are control, FADM, and FADM-WJ-MSCs groups during 21 days, respectively (scale bars = 100 μm, 10×; insets: 200 μm, 20×).

The results of the histopathological evaluation of the skin biopsies from the wound sites are presented in Figure 6. As shown, the inflammatory cells decreased with the number of days post-surgery in all experimental groups. On day 7, the number of inflammatory cells was higher in the control group compared to the FADM and FADM-WJMSC groups. Similarly, on day 14 post-surgery, the number of inflammatory cells was higher in the control group than the FADM group and the FADM group than the FADM-WJMSC group. The same results were also observed on day 21 post-surgery, as the lowest number of inflammatory cells was observed in the FADM-WJMSC group. Furthermore, the histopathological results indicate that neovascularization was higher in the FADM and FADM-WJMSC groups on days 14 and 21 post-surgery. Finally, the formation of hair follicles and sweat glands and the establishment of the neoepidermal layer was observed on day 21 post-surgery in the FADM and FADM-WJMSC groups. Fully completed wound re-epithelialization without inflammation was seen in the FADM and FADM-WJMSC groups. 

## 4. Discussion

The transplantation of allogeneic or xenogeneic WJ-MSCs has been shown to improve wound healing in various rat models of skin defects, including burn injuries and full-thickness defects. It is shown that MSCs, with their anti-inflammatory and immunomodulatory properties, promoted wound healing through stem cell proliferation (leading to wound coverage) and re-epithelialization. Acellular matrices have also been used in combination with MSCs to facilitate wound healing. Here, we used FADM to immobilize WJ-MSCs at wound sites.

Previous studies have indicated the potential of MSCs in the healing of full-thickness wounds in pre-clinical studies. In this regard, Abd-Allah and colleagues reported that MSCs derived from the placenta promoted the healing of skin wounds in a rat model of full-thickness wounds [26]. Hamra et al., also showed that MSCs accelerate wound closure in an experimental animal model [27]. A study by Qi et al., showed that the intradermal injection of MSCs promoted wound healing while reducing tissue fibrosis in a mouse model of full-thickness wounds [28]. The beneficial effects of MSCs from various sources, including Wharton’s jelly, have been indicated in various experimental and animal studies [29,30]. Nazempour and colleagues also reported that the use of allogeneic WJ-MSCs induced the healing of burn injuries in animal models [31]. 

The healing property of MSCs in wounds is attributed to their anti-inflammatory role, which is mostly conducted through their secretome [30]. MSCs from various sources express high levels of anti-inflammatory cytokines and, thus, reduce inflammatory markers and increase anti-inflammatory markers at the wound site [32]. MSCs release anti-inflammatory and immunomodulatory factors through their secretome. The secretome of MSCs is rich in extracellular vesicles (EVs) that reduce the expression of IL-1, IL-6, and TNF-α and increase the expression of IL-10 and ATP [33]. MSCs have been shown to inhibit the infiltration of inflammatory cells and increase the expression of collagen types I and III. Therefore, MSCs promote the healing of wounds by inducing keratinization and vascularization, as well as reducing inflammation [34]. 

Despite the healing function of MSCs at the wound site, the systemic or local administration of MSCs may lead to the loss of MSCs. In this regard, researchers have tried to immobilize MSCs at the wound site to induce effective wound closure. Wang et al., used collagen-binding peptides to induce the homing of MSCs at the wound site [35]. Some researchers also showed that nanofiber scaffolds could be used to immobilize MSCs with promising results [36,37,38,39]. As scaffolds from acellular matrices have attracted much attention in the treatment of degenerative diseases, their use has been proposed to improve wound healing. In a pre-clinical study, Li et al., showed that tilapia-skin ADM promoted wound healing by inducing angiogenesis and epithelialization [14]. In addition, greater skin wound healing was observed in rabbit models when ADM was used [40]. The beneficial effects of ADM in wound healing were also observed in other studies of animal models [41,42].

Currently, several human-derived ADMs, known as dermal substitutes (i.e., Alloderm and Dermalogen), are available to improve the wound healing of affected patients [43]. However, the use of these dermal substitutes is limited due to limitations regarding access to the source, morbidity at the donor site, the requirement for surgical intervention, and a prolonged period of recovery. An alternative approach is the use of xenograft ADMs, derived from rabbit [44], bovine [45], porcine [46], or sheep [47]. In our previous study, we reported that ADM from bovine could be efficiently used as a wound dresser in a rat model of full-thickness wounds. We found that the xenograft ADM improved the healing of wounds by up to 38% [25]. 

In this study, we used xenograft ADM derived from fetal bovine in combination with WJ-MSCs to improve the healing of the rat model of full-thickness wounds. Our results showed that the use of xenograft ADM, in combination with WJ-MSCs, not only significantly improved wound healing but no inflammation or immunological reactions were observed, and no specific growth factors were used. In our previous work, we also showed that the use of FADM induced neovascularization and skin regeneration while no inflammation was observed [25]. Here, we also showed that FADM-WJ-MSCs express a higher level of antigenic factors when cultured in vitro for 7 days. Several studies have reported the effects of MSCs in combination with ADM to improve wound healing. Wang et al., recellularized ADM with MSCs from bone marrow to improve wound healing in a murine model. ADM-MSCs promoted increased neovascularization and skin regeneration when compared to ADM or MSC alone [48]. A combination of dermal matrix and human MSCs significantly improved wound healing while reducing inflammatory responses in diabetic mice [49]. In the study by Orbay et al., better vascularization and collagen quantity were observed when MSCs from adipose tissue were used with ADM [50]. It is shown that the use of MSCs-ADM could also recruit endothelial cells to the injured/wound site, thereby facilitating vascularization and tissue granulation [51]. Another study also reported that MSC-ADM accelerated collagen deposition and decreased wound bed in an animal model of burn injury [52]. 

In our study, the wound closure was 94.1% and 100% in the ADM-WJMSC group after 14 and 21 days of transplantation, respectively. The wound closure was also 88.2% and 91.6% in the ADM group after 14 and 21 days of transplantation, respectively. This shows that ADM was able to improve wound healing, and the use of MSCs in combination with ADM also further accelerated wound closure. In the study by Nazempour et al., the wound closure was 87.6% in the ADM-WJMSC group after 21 days. This may show the better healing process of our construct in wound healing. However, we used animals with full-thickness skin defects, while Nazempour et al., used animals with burn injuries, making it difficult to compare the results of the studies [31]. Meanwhile, both studies indicated the potential of xenograft ADM in combination with MSCs in skin regeneration.

In the present study, we used an animal model of full-thickness defects to evaluate the healing properties of FADM-WJ-MSCs. However, it should be noted that the healing process of wounds in rats differs from human wounds. While in humans, the healing is based on re-epithelialization, in rats, the healing is also based on contraction mediated by panniculuscarnosus, which is removed through skin debridement [31]. In addition, the optimal number of MSCs required for skin regeneration was small (2 × 10^6^ cells for each matrix), which cannot be generalized to human patients. Moreover, this study evaluated the healing process for three weeks while longer times might be necessitated. On the other hand, we aimed to use ADM from fatal bovine as a xenograft matrix, and this matrix was used in the rat model of full-thickness defects. Finally, evaluating the tissue-specific gene expression and the immunohistochemical assessments of the generated skin could better support the findings and help to interpret the data.

## 5. Conclusions

In conclusion, our data showed that FADM-WJ-MSCs improved the healing of full-thickness wounds when compared to the control group. This demonstrates the skin regeneration potential of xenograft acellular matrix as a natural scaffold in combination with WJ-MSCs. It seems that the acellular xenograft matrix promoted the proliferation and differentiation of WJ-MSCs while increasing new granulation tissue formation, collagen synthesis, neovascularization, and epithelialization at the wound site. In addition, the fabricated construct showed no immunological responses. Therefore, FADM-WJ-MSCs may provide a novel therapeutic option to repair deep wounds. Besides the promising results of this study, further in-depth clinic opathological studies are required to prove the applicability of such matrices in pre-clinical as well as clinical settings.

## Figures and Tables

**Table 1:** The primer sequences.

Gene Name	Accession Number	Sequence	Product Size (bp)
*Β-actin*	NM_007393.5	F:5′ CTTCTTGGGTATGGAATCCTG	96
		R:5′ GTGTTGGCATAGAGGTCTTTAC	
*VEGFa*	NM_001110268.1	F:5′ TCGCTCCTCCACTTCTGAGG	73
		R: 5′ GGCCATTACCAGGCCTCTTC	
*bFGF*	NM_008006.2	F:5′CCGGTCACGGAAATACTCCA	89
		R:5′ CCTTCTGTCCAGGTCCCGTT	
*IL-1β*	NM_000576	F:5′ CCACAGACCTTCCAGGAGAATG	84
		R:5′GTGCAGTTCAGTGATCGTACAGG	

## Data Availability

The data sets used and/or analyzed forthis study are available from the corresponding author.
